# Kinetic Modelling and Test–Retest Reproducibility for the Dopamine D_1_R Radioligand [^11^C]SCH23390 in Healthy and Diseased Mice

**DOI:** 10.1007/s11307-020-01561-1

**Published:** 2020-11-11

**Authors:** Daniele Bertoglio, Jeroen Verhaeghe, Alan Miranda, Leonie Wyffels, Sigrid Stroobants, Celia Dominguez, Ignacio Munoz-Sanjuan, Mette Skinbjerg, Longbin Liu, Steven Staelens

**Affiliations:** 1 Molecular Imaging Center Antwerp (MICA), University of Antwerp, Wilrijk, Belgium; 2 Department of Nuclear Medicine, Antwerp University Hospital, Edegem, Belgium; 3 CHDI Management/CHDI Foundation, Los Angeles, CA USA

**Keywords:** Dopamine receptor, D_1_R, SCH23390, Preclinical imaging, Test-retest, Kinetic modelling, Huntington’s disease, Mouse, Q175DN

## Abstract

**Purpose:**

Our aim in this study was to compare different non-invasive pharmacokinetic models and assess test–retest reproducibility of the radioligand [^11^C]SCH23390 for the quantification of dopamine D_1_-like receptor (D_1_R) in both wild-type (WT) mice and heterozygous (HET) Q175DN mice as Huntington’s disease (HD) model.

**Procedures:**

Adult WT (*n* = 9) and HET (*n* = 14) mice underwent a 90-min [^11^C]SCH23390 positron emission tomography (PET) scan followed by computed tomography (CT) to evaluate the pharmacokinetic modelling in healthy and diseased conditions. Additionally, 5 WT mice and 7 HET animals received a second [^11^C]SCH23390 PET scan for test–retest reproducibility. Parallel assessment of the simplified reference tissue model (SRTM), the multilinear reference tissue model (MRTM) and the Logan reference tissue model (Logan Ref) using the striatum as a receptor-rich region and the cerebellum as a receptor-free (reference) region was performed to define the most suitable method for regional- and voxel-based quantification of the binding potential (*BP*_ND_). Finally, standardised uptake value ratio (SUVR-1) was assessed as a potential simplified measurement.

**Results:**

For all models, we measured a significant decline in dopamine D_1_R density (*e.g.* SRTM = − 38.5 ± 5.0 %, *p* < 0.0001) in HET mice compared to WT littermates. Shortening the 90-min scan duration resulted in large underestimation of striatal *BP*_ND_ in both WT mice (SRTM 60 min: − 17.7 ± 2.8 %, *p* = 0.0078) and diseased HET (SRTM 60 min: − 13.1 ± 4.1 %, *p* = 0.0001). Striatal *BP*_ND_ measurements were very reproducible with an average test–retest variability below 5 % when using both MRTM and SRTM. Parametric *BP*_ND_ maps generated with SRTM were highly reliable, showing nearly perfect agreement to the regional analysis (*r*^2^ = 0.99, *p* < 0.0001). Finally, SRTM provided the most accurate estimate for relative tracer delivery *R*_1_ with both regional- and voxel-based analyses. SUVR-1 at different time intervals were not sufficiently reliable when compared to *BP*_ND_ (*r*^2^ < 0.66).

**Conclusions:**

Ninety-minute acquisition and the use of SRTM for pharmacokinetic modelling is recommended. [^11^C]SCH23390 PET imaging demonstrates optimal characteristics for the study of dopamine D_1_R density in models of psychiatric and neurological disorders as exemplified in the Q175DN mouse model of HD.

**Supplementary Information:**

The online version contains supplementary material available at 10.1007/s11307-020-01561-1.

## Introduction

Dopamine D_1_-like receptors (D_1_R) are post-synaptic G protein-coupled receptors widely distributed in the central nervous system [1]. They are primarily expressed in the caudate and putamen nucleus with lower levels in limbic and cortical structures [2, 3]. Under physiological condition, dopamine D_1_R are involved in the modulation of the reward system, motor control and spatial working memory [4, 5]. However, alterations in dopamine release and dopamine D_1_R have been associated with the phenotype of different neurological and neuropsychiatric disorders, including Parkinson’s disease [6], schizophrenia [7], drug addiction [5, 8] and Huntington’s disease (HD) [9].

The radioligand [^11^C]SCH23390 ((R)-(+)-7-chloro-8-hydroxy-3-methyl-1-phenyl-2,3,4,5-tetrahydro-1H-3-benzazepine) [10, 11], similar to [^11^C]NNC-112 (8-chloro-7-hydroxy-3-methyl-5-(7-benzofuranyl)-2,3,4,5-tetrahydro-1H-3-benzazepine) [12, 13], is one of the most commonly employed radiotracers for non-invasive *in vivo* studies of dopamine D_1_R using positron emission tomography (PET) imaging.

The value of [^11^C]SCH23390 as a radiotracer to measure dopamine D_1_R using PET imaging in the putamen and the caudate nucleus has been largely demonstrated in clinical settings. In larger animals and humans, [^11^C]SCH23390 is commonly quantified using a 50–90-min dynamic PET scan with reference region-based kinetic modelling with either the simplified reference tissue model (SRTM) or the multilinear reference tissue model (MRTM) given their high test–retest reliability [14–18]. Nonetheless, kinetic modelling and test–retest reproducibility of [^11^C]SCH23390 in mice has not yet been investigated, an important limitation for its application to preclinical drug development. Indeed, dopamine D_1_R PET imaging is a potential phenotypical readout for therapeutic efficacy in neurological and neuropsychiatric disorders. For instance, dopamine D_1_R is markedly reduced in individuals with HD, as demonstrated *in vivo* using [^11^C]SCH23390 PET imaging [19–22]. This phenotype was also confirmed *in vitro* in the transgenic R6/2 and BACHD mouse models of HD using [^3^H]SCH23390 autoradiography [23] as well as *in vivo* in the knock-in Q175DN mouse model of HD using [^11^C]NNC-112 PET imaging [24]. Since the performance of a radioligand can vary with receptor density, we focused on the methodological characterisation of [^11^C]SCH23390 PET imaging using both wild-type (WT) mice as well as heterozygous (HET) Q175DN littermates [25, 26]. Our aims in the present study were threefold: firstly, investigate the capability of [^11^C]SCH23390 PET imaging to quantify dopamine D_1_R changes in Q175DN mice; secondly, compare radioligand performance, including time stability of outcome parameters, following regional- and voxel-based kinetic modelling using three different reference-based methods in both genotypes and assess possible semi-quantitative approaches and thirdly, measure the test–retest reproducibility of [^11^C]SCH23390 PET imaging in both genotypes.

## Materials and Methods

### Animals

Adult 10-month old heterozygous (HET, *n* = 14) male knock-in Q175DN mice (C57BL/6J background and same disease progression as the parental Q175 model [25, 26] with the removal of the neo-cassette used for the insertion of the expanded CAG sequence) and age-matched wild-type (WT, *n* = 9) littermates from Jackson Laboratories (Bar Harbour, Maine, USA) were included in the study. Given the sporadic congenital portosystemic shunt occurring in C57BL/6J mice [27], all animals were screened at Jackson Laboratories before shipment in order to avoid this variable as a confounding factor. Upon arrival, animals were group-housed in individually ventilated cages under a 12-h light/dark cycle in a temperature- and humidity-controlled environment. Food and water were provided *ad libitum* and more than one week of habituation was allowed before the start of the procedures. [^11^C]SCH23390 PET imaging was performed for all animals (HET, *n* = 14; WT, *n* = 9) for evaluation of the pharmacokinetic modelling in both healthy and disease mouse brains. For assessment of the [^11^C]SCH23390 test–retest reproducibility, 7 HET Q175DN and 5 WT littermates underwent a second [^11^C]SCH23390 PET scan 5.6 ± 1.6 days following the first scan.

### Radioligand Synthesis

[^11^C]SCH23390 synthesis was performed on an automated synthesis module (Carbosynthon I, Comecer, The Netherlands) based on the one-pot strategy [11] *via* common *N*-methylation of the desmethyl precursor. Briefly, [^11^C]MeI was added to a precooled (− 20 °C) reaction vessel containing *N*-desmethyl-SCH23390 (1.0 mg ± 10 %) and aqueous NaOH (1 M, 5 μl) in anhydrous DMF/DMSO (ratio 50/50, 300 μl) at room temperature. The reaction lasted for 8 min at 50 °C to synthesise [^11^C]SCH23390. The product was subsequently collected using a reverse phase semi-preparative HPLC column (Phenomenex Luna C18, 250 × 10 mm, 10 μm) with a biocompatible mobile phase (NaOAc 0.05 M pH 5.5/EtOH 96 %, 50/50, v/v) at a flow rate of 3.0 ml/min. Finally, the collected product was diluted (1 in 5) with saline solution through a sterile membrane filter in order to obtain an intravenously injectable solution. The radiochemical purity of the produced [^11^C]SCH23390 was determined using an isocratic HPLC method (Phenomenex Luna C18, 150 × 4.6 mm, 5 μm) with NaOAc 0.05 M pH 5.5/ACN, 70/30 (v/v) as a mobile phase, flow rate 1 ml/min and UV absorption at 280 nm. Molar activity at the end of the synthesis was 72.4 ± 4.7 GBq/μmol, with an average radiochemical purity greater than 99 %.

### PET Acquisition and Reconstruction

MicroPET/computed tomography (CT) images were acquired using two virtually identical Siemens Inveon PET/CT scanners (Siemens Preclinical Solution, Knoxville, USA). Animal preparation was performed as previously described [28, 29]. A bolus of radioligand was injected using an automated pump (Pump 11 Elite, Harvard Apparatus, USA) over a 12-s interval (1 ml/min) immediately after the start of the 90-min dynamic PET scan. [^11^C]SCH23390 was injected in a trace dose with WT mice receiving an average of 1.18 ± 0.28 μg/kg and HET littermates an average of 1.44 ± 0.37 μg/kg (*p* = 0.17) keeping the cold mass within 2.0 μg/kg to avoid potential mass effect. On the scan day, body weight was 30.9 ± 2.2 g and 26.9 ± 1.0 g for the WT and HET mice (*p* = 0.0015), respectively, with an injected activity of 4.6 ± 0.9 MBq for WT animals and 5.5 ± 1.5 MBq for HET Q175DN mice (*p* = 0.19). A significant reduction in body weight in this animal model of HD is commonly observed starting at 6 months of age [30, 31]; however, since we are performing dynamic acquisition and pharmacokinetic modelling, alterations in body weight are taken into account, and therefore, they were not expected to affect the quantification.

PET data were acquired in a list mode format and followed by a 10-min 80 kV/500 μA CT scan performed on the same gantry for attenuation correction and coregistration purposes. One WT animal received an injection that extravasated for the retest scan; therefore, it was omitted from the test–retest analysis. Acquired PET data were histogrammed and reconstructed into 39 frames of increasing length (12 × 10 s, 3 × 20 s, 3 × 30 s, 3 × 60 s, 3 × 150 s and 15 × 300 s) using a list mode iterative reconstruction with proprietary spatially variant resolution modelling in 8 iterations and 16 subsets of the 3D ordered subset expectation maximisation (OSEM 3D) algorithm [32]. Normalisation, dead time and CT-based attenuation corrections were applied. PET image frames were reconstructed on a 128 × 128 × 159 grid with 0.776 × 0.776 × 0.776 mm^3^ voxels.

### Image Analysis and Processing

Image analysis and processing of the PET data were performed in PMOD 3.6 software (Pmod Technologies, Zurich, Switzerland). Based on our previous observation that the use of magnetic resonance imaging (MRI) templates for spatial normalisation and VOI definition improves the accuracy of the regional quantification of PET data with focal uptake (as we previously investigated with the radioligand [^18^F]MNI-659 for phosphodiesterase 10A [31]), an MRI template for each genotype was obtained from another independent cohort of age-matched Q175DN WT (*n* = 6) and HET (*n* = 6) mice. WT and HET MR images were rigidly aligned to the space of the first animal and averaged to generate genotypic-specific MR templates. Since all animals were aligned to the same animal, both MRI templates are in the same space. PET registration was achieved by the rigid spatial normalisation of the individual CT images to the MR templates and then apply the same rigid transformation to the PET images. All images were visually checked for accuracy following spatial transformation. The volumes of interest (VOIs) were manually delineated on the genotype-specific MRI templates, and regional time–activity curves (TACs) were extracted for the striatum and whole cerebellum in order to perform kinetic modelling. No volumetric difference in brain structures was observed between the WT and HET MRI templates. The final volumes were as follows: the striatum 0.0215 cm^3^ for WT and 0.0208 cm^3^ for HET mice, while the cerebellum was the same for both genotypes (0.0507 cm^3^). The former was considered the receptor-rich region, while the latter was used as the receptor-free region [33]. Cortical structures were not considered given fivefold lower receptor density and low selectivity over serotoninergic 5-HT_2A_ receptors [18].

### Kinetic Modelling

We measured the non-displaceable binding potential (*BP*_ND_) analysing 3 different pharmacokinetic models. We compared the SRTM [34], the MRTM [35] and the Logan reference tissue model (Logan Ref) [36] in order to determine the most appropriate for estimation of [^11^C]SCH23390 *BP*_ND_ in the brain of both healthy WT animals and diseased HET Q175DN mice. When applying SRTM and MRTM, the relative tracer delivery *R*_1_ was also measured, while for the Logan Ref, the linear phase (t^*^) was fixed at t^*^ = 15 min with *k*_2_’ derived with MRTM. MRTM-based *k*_2_’ was preferred over the SRTM-based *k*_2_’ given the lower % standard error (SE) of the *k*_2_’ estimation observed with the former model (3.3 ± 0.3 % SE) compared to the latter (5.6 ± 1.9 % SE).

The relative performance of each model to fit the regional PET data was assessed by calculating the goodness-to-fit of the models using the Akaike Information Criterion (AIC) [37].

Time stability of the estimated striatal *BP*_ND_ (and *R*_1_ if applicable) was analysed for each investigated model by repeatedly excluding the last 5 min of PET acquisition from 90 min down to 45 min. The 90-min *BP*_ND_ and *R*_1_ were considered the reference outcome, and all the values obtained with shorter acquisitions were compared to the 90-min values. Variation in the estimation of *BP*_ND_ based on a shorter acquisition was considered acceptable only if the average percentage difference was lower than 10 % with an inter-individual standard deviation below 5 % when compared to the 90-min PET acquisition as previously applied [28, 29].

Parametric *BP*_ND_ maps were generated using SRTM [38], MRTM [35] and Logan reference tissue model [36] with the *k*_2_’ as calculated with MRTM. SRTM2 [39] and MRTM2 [35] were also explored. Nonetheless, they did not improve the reliability of the parametric maps as SRTM was already accurate and MRTM2 was still presenting failed voxels; thus, we report SRTM and MRTM in order to investigate the agreement between parametric maps and regional analysis. Besides, parametric *R*_1_ maps were generated using SRTM and MRTM. For all models, the striatum was considered the receptor-rich region, while the cerebellum represented the receptor-devoid region (reference region). Parametric images were cropped using the brain mask of the MRI template, represented as group averages and overlaid onto the genotype-specific MRI templates for anatomical reference.

Additionally, we wanted to relate striatal *BP*_ND_ values obtained using the regional- and voxel-based analyses to determine the reliability of the parametric maps within each pharmacokinetic model. To this end, following the generation of the parametric *BP*_ND_ maps, we applied the VOI generated for the regional analysis in order to average the *BP*_ND_ of each striatal voxel. Next, we compared striatal *BP*_ND_ values calculated using the voxel-based maps and regional analysis to assess their agreement within each pharmacokinetic model.

Finally, we explored the applicability of a simplified approach for the quantification of striatal [^11^C]SCH23390 binding by measuring the ratio of the striatal standardised uptake values (SUV) over the cerebellar SUV (denoted as SUVR) based on the scan intervals 40–60 min as well as 70–90 min. The resulting measurement, SUVR-1, was compared to *BP*_ND_.

### Statistical Analysis

All the data were normally distributed as assessed with the Shapiro–Wilk test; therefore, parametric analyses were performed. Unpaired *T-*tests were performed to compare scan parameters, *BP*_ND_, SUVR-1 and *R*_1_ between genotypes during both VOI-based and voxel-based analyses. Given the sample size of WT mice in the test–retest study (*n* = 4), a comparison of the test–retest scan parameters was performed using paired *T*-test under the assumption of normality in the distribution. All correlations between variables were investigated with Pearson’s correlation tests and linear regression analyses. Bland–Altman plots, reported as bias and 95 % limits of agreement (1.96 × SD), were used to assess agreement between test–retest scans in the estimation of striatal *BP*_ND_ and *R*_1_. In addition, the reproducibility of the test–retest data was determined by the intraclass correlation coefficient (ICC), relative test–retest variability (TRV) and absolute TRV (aTRV). A mixed-model reliability analysis for absolute agreement was performed for the assessment of ICC with genotype included as the fixed effect in the model.

TRV was calculated as follows:

$$ \mathrm{TRV}=\frac{\mathrm{retest}\ \mathrm{value}-\mathrm{test}\ \mathrm{value}}{\overline{\left(\mathrm{retest}\ \mathrm{value}+\mathrm{test}\ \mathrm{value}\right)}}\ \mathrm{x}\ 100\%, $$

while aTRV was measured as follows:

$$ \mathrm{aTRV}=\frac{\mid \mathrm{retest}\ \mathrm{value}-\mathrm{test}\ \mathrm{value}\mid }{\overline{\left(\mathrm{retest}\ \mathrm{value}+\mathrm{test}\ \mathrm{value}\right)}}\ \mathrm{x}\ 100\%. $$

Finally, the mean ± standard deviation (SD) of the intra-animal coefficient of variation (COV) was calculated as follows:

$$ {\mathrm{COV}}_G=\frac{1}{N}{\sum}_i^N\frac{{\mathrm{SD}}_i^G}{{\overline{x}}_i^G}, $$

where *G* represents the group, *N* is the number of animals in the group, ${\overline{x}}_i^G$and ${\mathrm{SD}}_i^G$ are respectively the mean and standard deviation of the test and retest values for animal *i*. All aforementioned statistical analyses were performed using GraphPad Prism (v8.4) statistical software except for ICC, calculated in JMP Pro 14 software (SAS Institute Inc., USA). The effect size *d*, determined using G^∗^Power software (http://www.gpower.hhu.de/), was calculated using the mean and variance of each experimental group (WT and HET). Data are represented as mean ± SD, unless specified otherwise. All tests were two-tailed and significance was set at *p* < 0.05.

## Results

### Striatal [^11^C]SCH23390 *BP*_ND_ Quantification

**Fig. 1.**
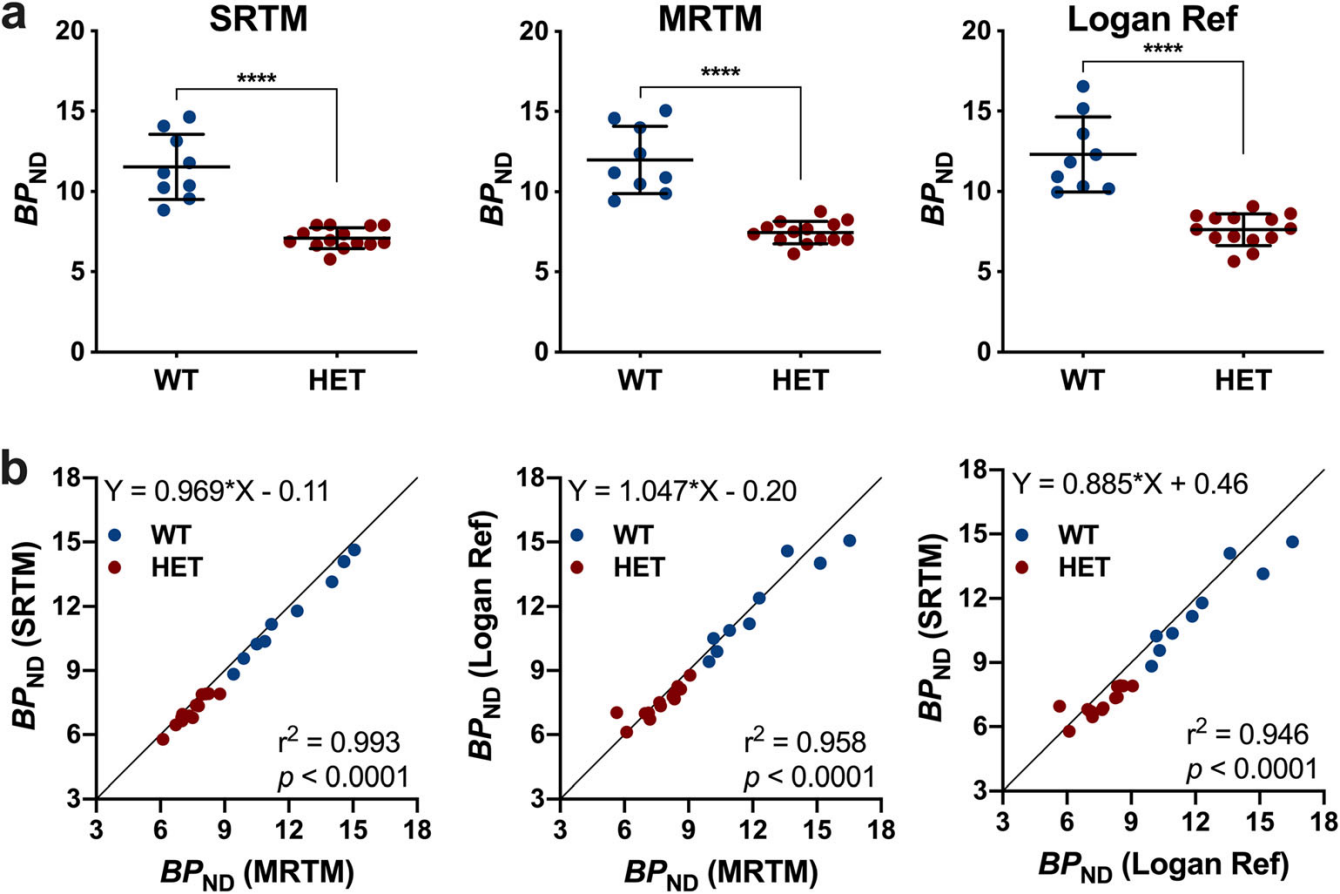
Striatal [^11^C]SCH23390 *BP*_ND_ quantification based on 90 min acquisition in WT and HET Q175DN mice. **a** [^11^C]SCH23390 *BP*_ND_ was significantly reduced in HET mice compared to WT littermates using SRTM, MRTM and Logan Ref. **b** SRTM and MRTM displayed a nearly perfect agreement in the estimation of *BP*_ND_ in both WT and HET Q175DN mice. Solid black line represents the identity line. ^****^p < 0.0001. WT, *n* = 9; HET, *n* = 14. *BP*_ND_ non-displaceable binding potential, *WT* wild-type, *HET* heterozygous.

Striatal [^11^C]SCH23390 *BP*_ND_ in HET Q175DN mice at 10 months of age was significantly reduced compared to WT littermates (Fig. 1a). Representative SUV time–activity curves of one WT and one HET Q175DN animal are shown in Suppl. Fig. 1 (see ESM). All investigated kinetic models displayed a comparable decline of approximately 38 % (range of − 37.9 to − 38.5 %). While SRTM and MRTM were characterised by a nearly perfect relationship in the estimation of striatal *BP*_ND_ (*r*^2^ = 0.99) (Fig. 1b), the Logan reference method showed larger variability (WT = 12.31 ± 2.34, HET = 7.62 ± 0.98, − 38.1 ± 5.7 %, *p* < 0.0001) compared to SRTM (WT = 11.54 ± 2.03, HET = 7.09 ± 0.65, − 38.5 ± 5.0 %, *p* < 0.0001) and MRTM (WT = 11.99 ± 2.10, HET = 7.44 ± 0.70, − 37.9 ± 5.0 %, *p* < 0.0001) (Table 1). Accordingly, the Logan Ref method resulted in a reduced effect size *d* (Table 1).

**Table 1.** Comparison of 3 reference kinetic models for estimation of striatal [^11^C]SCH23390 *BP*_ND_ in the striatum of WT and HET Q175DN mice based on 90-min acquisition

	*BP*_ND_ (SRTM)		*BP*_ND_ (MRTM)		*BP*_ND_ (Logan Ref)	
Genotype	Mean (SD)	COV (%)	Mean (SD)	COV (%)	Mean (SD)	COV (%)
WT	11.54 (2.03)	17.6 %	11.99 (2.10)	17.5 %	12.31 (2.34)	19.0 %
HET	7.09 (0.65)	9.1 %	7.44 (0.70)	9.4 %	7.62 (0.98)	12.9 %
Diff (%)	38.5 %		37.9 %		38.1 %	
Effect size *d*	*d* = 2.9		*d* = 2.9		*d* = 2.6	

*BP*_ND_ non-displaceable binding potential, *SRTM* simplified reference tissue model, *MRTM* multilinear reference tissue model, *Logan Ref* Logan reference tissue model, *Diff* genotypic difference, *SD* standard deviation, *COV* coefficient of variation, *WT* wild-type, *HET* heterozygous. WT, *n* = 9; HET, *n* = 14

The striatal AIC values indicated MRTM as the model with the best performance (lower value) in both WT (MRTM = 21.2 ± 10.2, SRTM = 30.2 ± 10.9) and HET (MRTM = 4.6 ± 13.7, SRTM = 26.2 ± 14.5) mice.

### Time Stability of the Striatal [^11^C]SCH23390 *BP*_ND_ Estimates

**Fig. 2.**
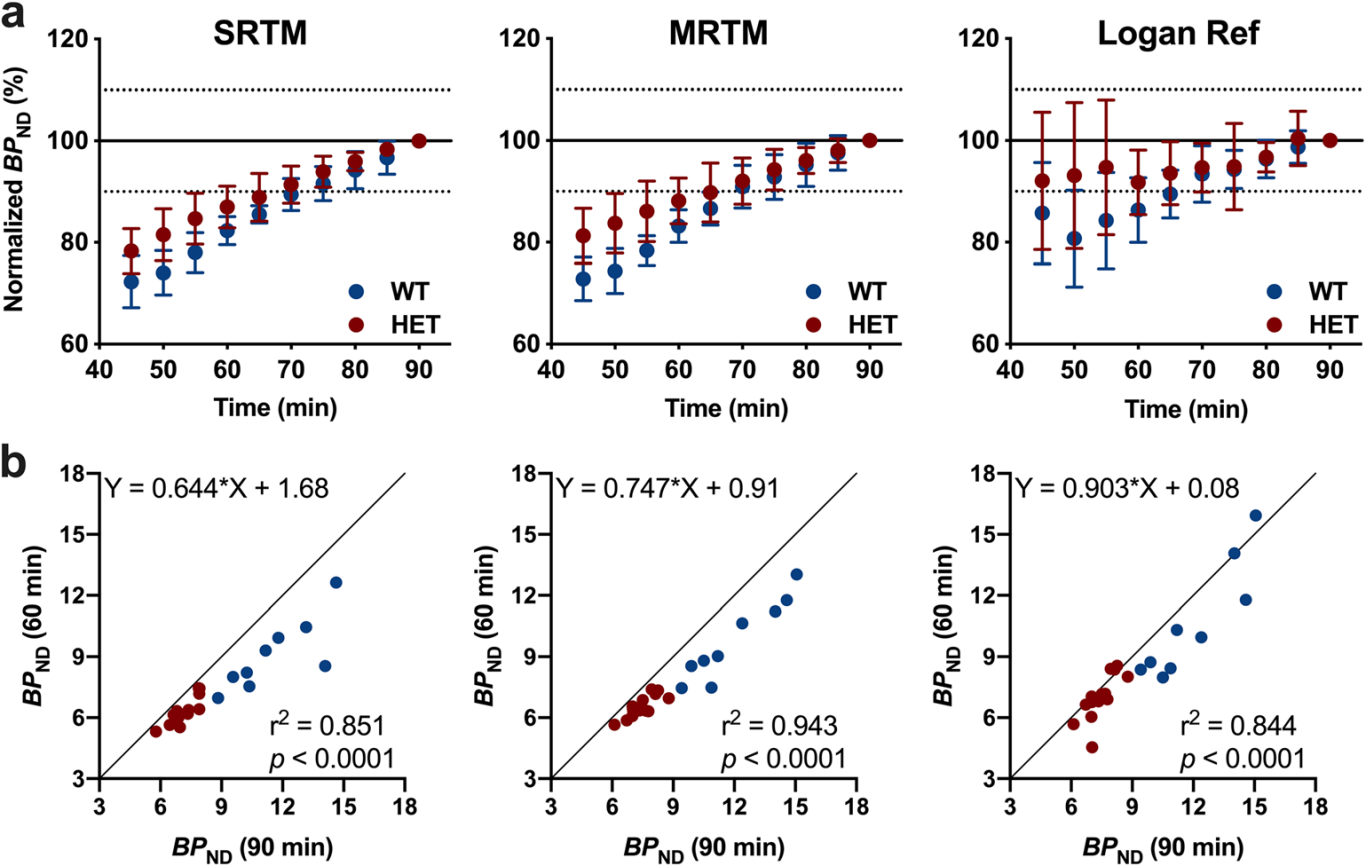
Time stability of the *BP*_ND_ estimates using different methods in the striatum of WT and HET Q175DN mice. **a***BP*_ND_ estimations were normalised to the values obtained during 90-min acquisition. **b** Correlation between striatal *BP*_ND_ using SRTM, MRTM and Logan Ref calculated based on 90-min and 60-min acquisition. Solid black line represents the identity line. WT, *n* = 9; HET, *n* = 14. *BP*_ND_ non-displaceable binding potential, *WT* wild-type, *HET* heterozygous.

Next, in order to assess the time stability of striatal *BP*_ND_, we investigated the effect of the duration of the PET acquisition on its estimation for all the kinetic models (SRTM, MRTM and Logan reference). As depicted in Fig. 2a, when normalising shorter scan durations to the 90-min *BP*_ND_ for each subject, a large underestimation of striatal *BP*_ND_ was introduced in both healthy WT mice and diseased HET Q175DN animals, with the largest variability observed using the Logan reference method. Reducing the acquisition time down to 60 min resulted in marked biases for both healthy WT mice (− 17.7 ± 2.8 % with SRTM, *p* = 0.0078; − 16.8 ± 3.2 % with MRTM, *p* = 0.0156 and − 13.7 ± 6.3 % with Logan reference, *p* = 0.0078) and diseased HET Q175DN animals (− 13.1 ± 4.1 % with SRTM, *p* = 0.0001; −11.8 ± 4.5 % with MRTM, *p* = 0.0001 and − 8.2 ± 6.3 % with Logan reference, *p* = 0.0006). The underestimation in *BP*_ND_ estimates when considering 60-min instead of 90-min acquisition can also be appreciated by the deviation from the identity line observed using SRTM (slope = 0.644), MRTM (slope = 0.747) and Logan Ref (slope = 0.903) (Fig. 2b). Consequently, all models displayed a reduced genotypic difference in striatal *BP*_ND_ (SRTM = − 30.4 ± 5.6 %, *p* < 0.0001; MRTM = − 33.0 ± 5.6 %, *p* < 0.0001 and Logan Ref = − 34.0 ± 7.7 %, *p* = 0.0003) when compared to the 90-min acquisition (Fig. 1a).

### Test–Retest Reproducibility of Striatal [^11^C]SCH23390 *BP*_ND_ Estimates

**Fig. 3.**
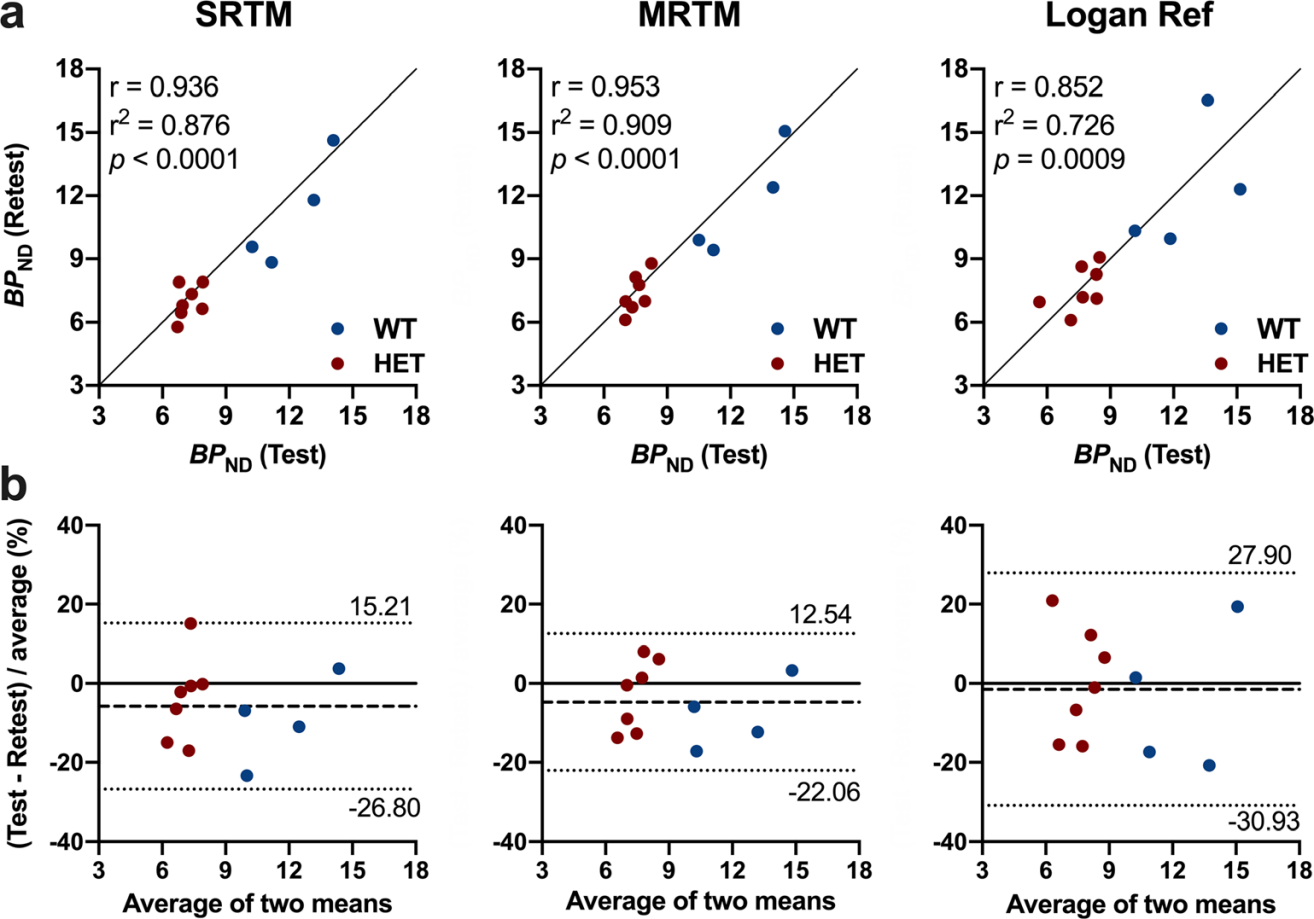
Test–retest reproducibility of [^11^C]SCH23390 *BP*_ND_ estimates derived by different methods based on 90-min acquisition in the striatum of WT and HET Q175DN mice. **a** Correlation between test and retest *BP*_ND_. **b** Bland–Altman plot to compare test–retest quantification of *BP*_ND_. The dashed line depicts the bias between the two scans, while the dotted lines represent the 95 % limits of agreement. WT, *n* = 4; HET, *n* = 7. *BP*_ND_ non-displaceable binding potential, *WT* wild-type, *HET* heterozygous.

For the test and retest scans, no significant methodological confounding factors were observed (Suppl. Table 1, see ESM). Striatal test–retest *BP*_ND_ measurements using SRTM, MRTM and Logan Ref are reported in Table 2. Overall, striatal [^11^C]SCH23390 *BP*_ND_ was reliable with the highest reproducibility using the MRTM and SRTM methods. For instance, the lowest aTRV values were measured with MRTM in both WT mice (SRTM = 5.6 %; MRTM = 4.8 % and Logan Ref = 7.4 %) and HET Q175DN animals (SRTM = 4.0 %; MRTM = 3.5 % and Logan Ref = 6.9 %) (Table 2). Accordingly, the combined (WT and HET) ICC values indicated high striatal [^11^C]SCH23390 *BP*_ND_ reproducibility with SRTM (0.748) and MRTM (0.810), while a low performance when using Logan Ref (0.457) (Table 2). The overall test–retest correlations displayed the highest agreement with MRTM (*r*^2^ = 0.91, *p* < 0.0001), followed by SRTM (*r*^2^ = 0.87, *p* < 0.0001) and the lowest for Logan Ref (*r*^2^ = 0.72, *p* = 0.0009) (Fig. 3a and Table 2). Similarly, the combined (WT and HET) Bland–Altman plots showed only negligible biases (SRTM = − 5.79 %, MRTM = − 4.76 % and Logan Ref = − 1.51 %), although the 95 % limits of agreement were relatively large for Logan Ref (− 30.9 % and 27.9 %) (Fig. 3b and Table 2).

**Table 2.** Test–retest reproducibility of striatal [^11^C]SCH23390 *BP*_ND_ estimates in WT mice and HET Q175DN littermates based on 90-min acquisition

Model	Test	Retest	TRV (%)	aTRV (%)	Bias (%)	ICC	*r*^2^
	Mean (sem)	Mean (sem)	Mean (sem)	Mean	Mean		
SRTM							
WT	11.8 (0.8)	11.2 (1.0)	4.6 (2.5)	5.6	− 5.79	0.748	0.876
HET	7.2 (0.2)	7.0 (0.3)	0.3 (2.3)	4			
MRTM							
WT	12.2 (1.9)	11.7 (2.6)	4.0 (1.9)	4.8	− 4.76	0.810	0.909
HET	7.5 (0.2)	7.3 (0.3)	0.0 (1.8)	3.5			
Logan Ref							
WT	12.3 (0.9)	12.3 (1.3)	2.1 (4.1)	7.4	− 1.51	0.457	0.726
HET	7.6 (0.4)	7.6 (0.4)	− 3.0 (2.9)	6.9			

*BP*_ND_ non-displaceable binding potential, *SRTM* simplified reference tissue model, *MRTM* multilinear reference tissue model, *Logan Ref* Logan reference tissue model, *sem* standard error of the mean, *TRV* test–retest variability, *aTRV* absolute TRV, *ICC* intraclass correlation coefficient, *WT* wild-type, *HET* heterozygous. WT, *n* = 4; HET, *n* = 7

### Parametric [^11^C]SCH23390 *BP*_ND_ Maps

**Fig. 4.**
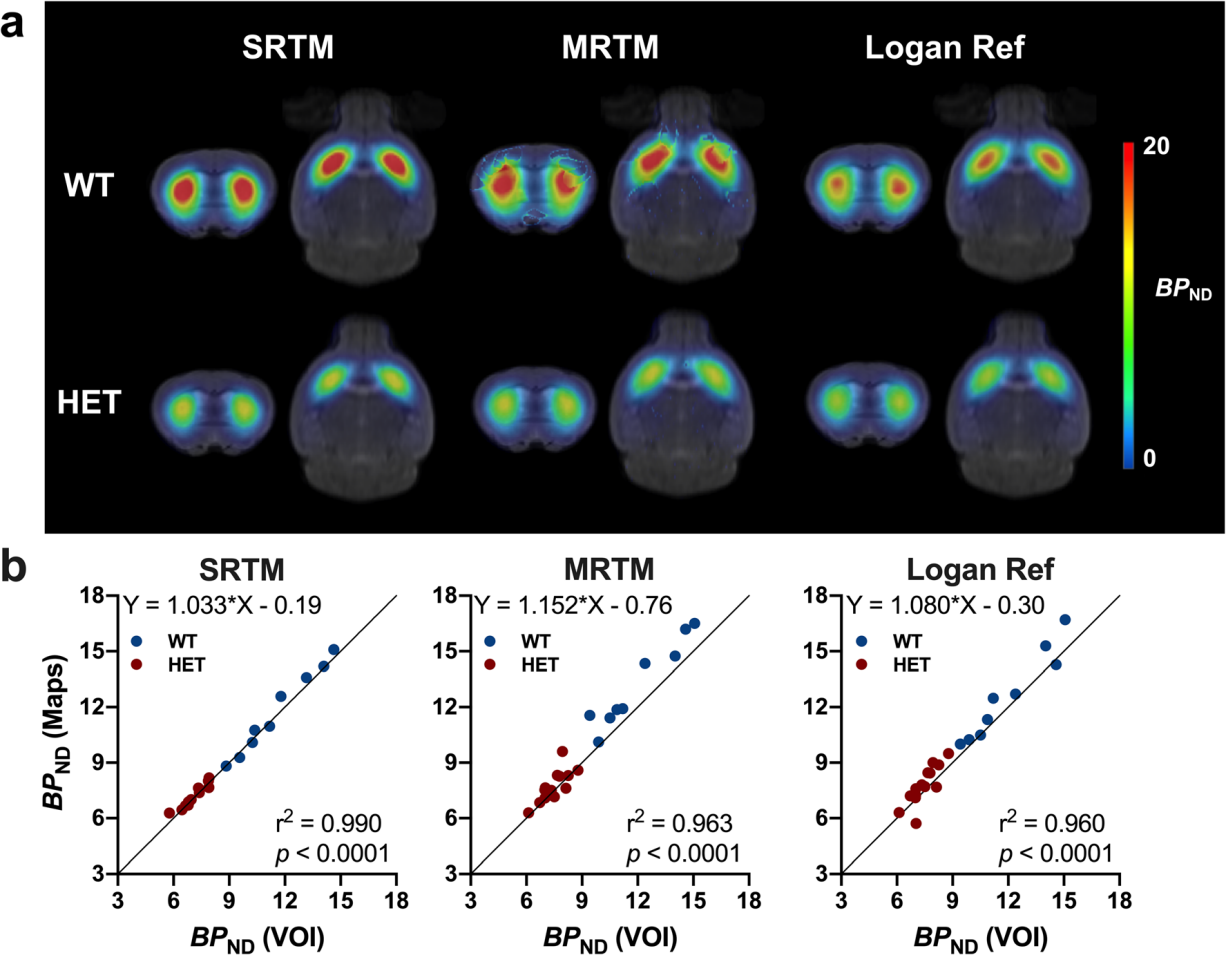
Average parametric [^11^C]SCH23390 *BP*_ND_ maps based on 90-min acquisition in WT and HET Q175DN mice. **a** Maps are generated using SRTM, MRTM and Logan Ref, and they are overlaid onto a genotype-specific MRI template for anatomical localisation. Parametric maps obtained with MRTM displayed many failed voxels randomly scattered across many animals. **b** Correlation between striatal *BP*_ND_ using SRTM, MRTM and Logan Ref calculated using regional (VOI) and voxel-based (maps) analyses. WT, *n* = 9; HET, *n* = 14. *BP*_ND_ non-displaceable binding potential, *WT* wild-type, *HET* heterozygous, *VOI* volume of interest.

Average voxel-based parametric *BP*_ND_ maps for WT mice and HET Q175DN animals are shown in Fig. 4a. Visually, parametric *BP*_ND_ maps generated with SRTM resulted in reliable maps showing a nearly perfect agreement to the regional analysis (*r*^2^ = 0.99, *p* < 0.0001) and no deviation from the identity line (slope = 1.03) (Fig. 4b). On the contrary, parametric *BP*_ND_ maps obtained using MRTM were characterised by many failed voxels randomly scattered across different animals (Fig. 4a). Besides, even though the MRTM-based striatal *BP*_ND_ values obtained using the parametric maps agreed well with the VOI-based analysis (*r*^2^ = 0.96, *p* < 0.0001), they deviated from the identity line (slope = 1.15) (Fig. 4b). The Logan reference method produced an agreement between striatal *BP*_ND_ values based on parametric maps and regional analysis similar to MRTM (*r*^2^ = 0.96, *p* < 0.0001) (Fig. 4b).

### Estimation of [^11^C]SCH23390 Relative Tracer Delivery *R*_1_

**Fig. 5:**
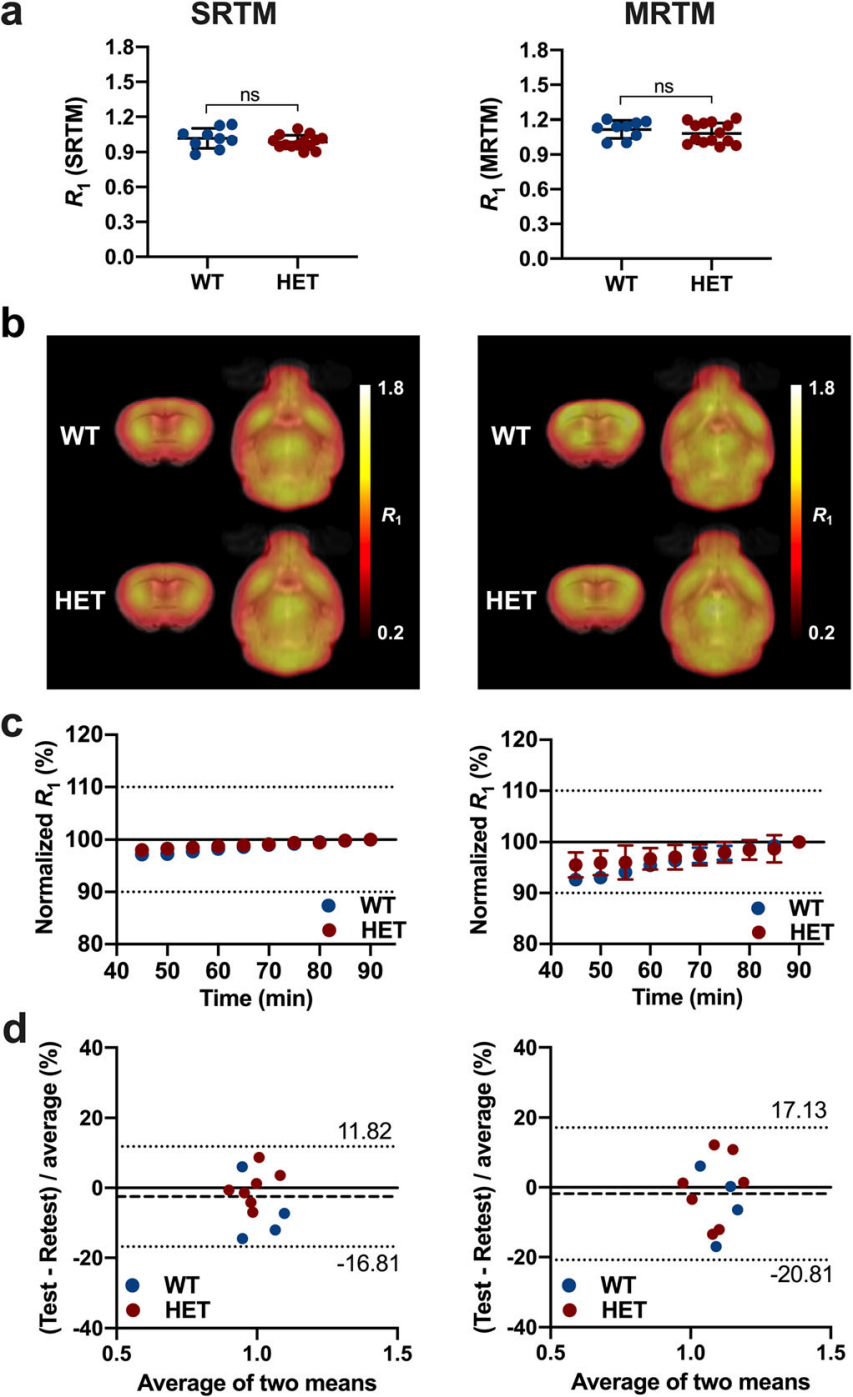
Estimation of [^11^C]SCH23390 relative tracer delivery *R*_1_ using SRTM and MRTM based on 90-min acquisition in WT and HET Q175DN mice. **a** [^11^C]SCH23390 *R*_1_ did not differ between WT and HET Q175DN mice. **b** Average parametric [^11^C]SCH23390 *R*_1_ maps overlaid onto a genotype-specific MRI template for anatomical localisation. **c** Time stability of the striatal *R*_1_ at different scan duration compared to the 90 min. (d) Bland–Altman plot between test–retest quantification of *R*_1_. The bias between the test and retest scans corresponds to the difference between the mean (dashed line) and *X*-axis (solid line). The dotted lines represent the 95 % limits of agreement. WT, *n* = 9; HET, *n* = 14. *ns* not significant, *WT* wild-type, *HET* heterozygous.

Finally, the relative tracer delivery *R*_1_ based on a 90-min acquisition was assessed (Fig. 5). [^11^C]SCH23390 *R*_1_ in the striatum of HET Q175DN mice at 10 months of age did not differ from WT littermates when using either SRTM (WT = 1.02 ± 0.08, HET = 0.98 ± 0.06, − 3.1 %, *p* = 0.299) or MRTM (WT = 1.11 ± 0.07, HET = 1.08 ± 0.09, − 2.9 %, *p* = 0.379) (Fig. 5a). Reliable parametric *R*_1_ maps could be generated for both WT and HET Q175DN mice as shown in Fig. 5b. Time stability of striatal *R*_1_ estimation using SRTM was excellent, with only a negligible bias for both WT (− 1.77 %) and Q175DN mice (− 1.27 %) even when considering an acquisition of 60 min compared to 90 min (Fig. 5c). Striatal *R*_1_ estimations based on MRTM were less stable in both WT (− 4.57 %) and HET Q175DN animals (− 3.26 %) (Fig. 5c). In addition, the Bland–Altman plot for the WT and HET mice combined resulted in a bias of only − 2.5 % (SRTM) and − 1.83 % (MRTM) with low 95 % confidence intervals using SRTM (− 16.8 % and 11.8 %) and moderate 95 % limits of agreement when using MRTM (− 20.8 % and 17.1 %) (Fig. 5d).

### Applicability of Simplified [^11^C]SCH23390 Measurements

Given the extensive 90-min dynamic acquisition recommended for [^11^C]SCH23390 *BP*_ND_ estimation, we explored the applicability of SUVR-1 based on the scan intervals 40–60 min as well as 70–90 min. As shown in Suppl. Fig. 2a (see ESM), HET mice displayed reduced SUVR-1 values when compared to WT mice (*p* < 0.01); however, the phenotypic difference was underestimated with both time intervals (SUVR-1_(40–60)_: − 26.7 %; SUVR-1_(70–90)_: − 29.6 %) compared to the − 38.5 % measured with *BP*_ND (SRTM)_. Additionally, SUVR-1 were not sufficiently reliable when compared to *BP*_ND_ (SUVR-1_(40–60)_: *r*^2^ = 0.609, *p* < 0.0001; SUVR-1_(70–90)_: *r*^2^ = 0.651, *p* < 0.0001) to represent a valid alternative to the dynamic acquisition (Suppl. Fig. 2b, see ESM).

## Discussion

Our study compared three reference region-based pharmacokinetic models to quantify [^11^C]SCH23390 PET imaging to determine the optimal methodology for striatal *BP*_ND_ and *R*_1_ estimation in the mouse brain. We also evaluated this radioligand in a diseased condition characterised by the reduction of dopamine D_1_R density in the Q175DN HD mouse model, which exhibits several HD phenotypic hallmarks [24–26, 31, 40], including impairment of the dopaminergic system [24, 41, 42]. Reduction of striatal dopaminergic D_1_ and D_2/3_ receptors has been largely documented in patients with premanifest and manifest HD compared to healthy controls [19–22, 43]. Here, we measured an *in vivo* dopamine D_1_R reduction of 38 % in HET Q175DN at 10 months of age using [^11^C]SCH23390, similar to the 34 % reduction previously reported at 9 months of age using [^11^C]NNC-112 [24]. The relevance of these findings is further substantiated by previous clinical reports describing a subtle reduction in presymptomatic patients [22] followed by a 35–40 % decline in D_1_R with the progression of the disease [19] as measured *in vivo* with [^11^C]SCH23390 PET imaging.

To assess [^11^C]SCH23390 kinetic modelling, we focused on three reference methods, namely, SRTM, MRTM and Logan reference, using the cerebellum as a reference region. When evaluating the VOI-based analysis, MRTM resulted in the best performance, as supported by the AIC, with SRTM being a valid alternative, while Logan Ref displayed the lowest accuracy in striatal *BP*_ND_ estimation. However, when conducting voxel-based analysis, *BP*_ND_ [^11^C]SCH23390 maps obtained with MRTM displayed failed voxels in nearly half of the WT animals in the proximity of high uptake structures as depicted in Suppl. Fig. 3 (see ESM). Additionally, MRTM and Logan Ref had similar suboptimal performances (*r*^2^ = 0.96 and *r*^2^ = 0.96, respectively), while SRTM was the most accurate (*r*^2^ = 0.99). Even though from a strictly VOI-based kinetic modelling perspective, MRTM proved to be slightly more accurate than SRTM; for studies where voxel-based parametric maps are also of interest, SRTM represents the optimal balance for both VOI- and voxel-based analyses. Previous studies investigating the kinetics in humans and baboons have reported using SRTM to estimate *BP*_ND_ [18, 34, 44, 45], while more recently, the simplified MRTM method, MRTM2, was applied in a human study [14]. Additionally, reported radioligand kinetics in humans and larger animals suggest a faster striatal washout when compared to mice, with PET scan acquisition ranging from 50 to 90 min [14–18].

Evaluation of time stability of striatal *BP*_ND_ showed that shortening the acquisition to less than 90 min led to large underestimations of striatal *BP*_ND_ in both healthy and diseased mice for all assessed kinetic models, which was expected given the slow washout of [^11^C]SCH23390 in the striatum, especially in healthy animals with higher receptor density. Thus, based on a 90-min acquisition, a scan duration of at least 80 min is necessary to accurately estimate striatal *BP*_ND_ in mouse brain. Longer scan acquisition might be potentially required to characterise even more precisely the dynamics of the receptor. Nonetheless, within the 90-min acquisition [^11^C]SCH23390 kinetics appeared to be stable and, due to the short half-life of the radioisotope (20.3 min), a longer acquisition may likely introduce noise hampering the quantification.

Since reliable quantification of the receptor density is fundamental to conduct pharmacological, interventional and longitudinal studies in animal models, we investigated both healthy WT and diseased HET Q175DN mice, in which the dopamine D_1_R density is reduced [24], to evaluate test–retest reproducibility of [^11^C]SCH23390 striatal *BP*_ND_ in mouse brain. *BP*_ND_ measurements had an extremely low test–retest variability with absolute TRV ranging from 0 to 11 % (on average below 6 %) for the WT and HET mice with both SRTM and MRTM. Striatal *BP*_ND_ quantification was also reproducible with both SRTM (ICC = 0.748) and MRTM (ICC = 0.810). [^11^C]SCH23390 test–retest reproducibility has not been assessed before in rodents, but human studies have reported high test–retest stability with ICC values ranging from 0.81 to 0.94 [14, 44, 46].

Estimation of striatal [^11^C]SCH23390 *BP*_ND_ recommends a 90-min dynamic acquisition. To avoid such extensive acquisition, we evaluated static time intervals quantified with SUVR-1 as an alternative since it has been previously reported a robust approach with other radioligands [47–49]. Nevertheless, it did not prove sufficiently reliable when compared to *BP*_ND_ with either time interval (40–60 min: *r*^2^ = 0.609; 70–90 min: *r*^2^ = 0.651). A possible reason for the lack of reliability may be associated to the noise levels in the cerebellum as a consequence of the low uptake in parallel to the rapid decay of the ^11^C radioisotope.

Finally, *R*_1_ could be reliably estimated using the SRTM and MRTM methods for both regional- and voxel-based approaches. Noteworthy, estimation based on SRTM was more reliable than MRTM with both regional- and voxel-based analyses. Striatal *R*_1_ estimation using SRTM was extremely stable down to 45-min acquisition for both WT mice (− 2.84 %) and HET Q175DN animals (− 1.98 %) compared to 90 min, with extremely good test–retest reproducibility (bias = 2.5 %).

In the brain, dopamine D_1_R are found mainly in the terminal structures of the dopaminergic system (striatum) with lower density in the cortical areas [3, 50], so dopamine D_1_R PET imaging could potentially be applied to cortical structures to study psychiatric disorders such as schizophrenia [51]. However, when considering the cerebral binding affinity, *in vitro* studies with SCH23390 reported a dissociation constant (*K*_D_) of 0.14–0.37 nM for dopamine D_1_R and *K*_D_ of 19.9–37 nM for 5-HT_2A_R in the rodent brain [18, 52–54]. Similarly, an *in vitro K*_D_ of 0.18 nM for dopamine D_1_R and *K*_D_ of 18 nM for 5-HT_2A_R have been described for NNC-112 [12, 18]. Thus, the selectivity of both [^11^C]SCH23390 and [^11^C]NNC-112 towards the serotoninergic 5-HT_2A_ receptors are negligible (*circa* 100-fold lower) compared to the dopamine D_1_R. Accordingly, *in vivo* PET studies in baboons and humans demonstrated that approximately a quarter of cortical *BP*_ND_ of [^11^C]SCH23390 and [^11^C]NNC-112 is driven by 5-HT_2A_R binding [18, 55, 56]. Although cortical selectivity has not been investigated in rodents, the combined contribution of dopamine D_1_R and 5-HT_2A_R to the cortical signal represents a shortcoming of the current dopamine D_1_R radioligands and should be addressed in future studies. Consequently, caution is warranted before interpreting cortical-binding changes with the currently available dopamine D_1_R radioligands. Without more selective radioligands, coinjection of a 5-HT_2A_R blocker and the radioligand might enable accurate quantification of D_1_R cortical binding *in vivo*, as previously postulated [56].

## Conclusion

We recommend a 90-min acquisition and the use of SRTM for pharmacokinetic modelling of [^11^C]SCH23390 in healthy and diseased mice in order to achieve reproducible values and reliable parametric *BP*_ND_ and *R*_1_ maps. Our findings demonstrate the utility of [^11^C]SCH23390 PET imaging for the study of dopamine D_1_R density in psychiatric and neurological disorders as exemplified in the Q175DN HD mouse model.

## Supplementary Information

ESM 1 (DOCX 3209 kb)
