# Supplementary material for: Kinetic Modelling and Test–Retest Reproducibility for the Dopamine D1R Radioligand [11C]SCH23390 in Healthy and Diseased Mice
Source: Mol Imaging Biol. 2020 Nov 11;23(2):208–19. doi: 10.1007/s11307-020-01561-1 (PMC7910372; doi:10.1007/s11307-020-01561-1)
Supplement: Supplementary file 1 — (DOCX 3209 kb) [file 11307_2020_1561_MOESM1_ESM.docx]

**Electronic Supplementary Material**

**Kinetic modelling and test-retest reproducibility for the dopamine D_1_R radioligand [^11^C]SCH23390 in healthy and diseased mice**

**Journal: Molecular Imaging and Biology**

Daniele Bertoglio^1^, Jeroen Verhaeghe^1^, Alan Miranda^1^, Leonie wyffels^1,2^, Sigrid Stroobants^1,2^, Celia Dominguez^3^, Ignacio Munoz-Sanjuan^3^, Mette Skinbjerg^3^, Longbin Liu^3^, Steven Staelens^1^

^1^Molecular Imaging Center Antwerp (MICA), University of Antwerp, Wilrijk, Belgium

^2^Department of Nuclear Medicine, Antwerp University Hospital, Edegem, Belgium

^3^CHDI Management/CHDI Foundation, Los Angeles, California, USA

**Correspondence to:**

Prof. Steven Staelens

Tel. +32 03265 2820

Email: [steven.staelens@uantwerpen.be](mailto:steven.staelens@uantwerpen.be)

**Supplementary Figures**

**Supplementary Fig. 1. Representative [^11^C]SCH23390 SUV time-activity curves in striatum and cerebellum of a representative WT and HET Q175DN animal.** STR = striatum, CB = cerebellum, WT = wild-type, HET = heterozygous.

**Supplementary Fig. 2. Assessment of striatal [^11^C]SCH23390 binding in WT and HET Q175DN mice. (a)** SUVR-1 [^11^C]SCH23390 measurement based on the time intervals 40-60 min (left) or 70-90 min (right) in WT and HET mice. **(b)** Relationship between *BP*_ND_ based on SRTM and SUVR-1 in the time intervals 40-60 min (left) and 70-90 min (right). Solid black line represents identity line. ***p* < 0.01. WT, *n* = 9; HET, *n* = 14. *BP*_ND_ = non-displaceable binding potential, SUVR = standardized uptake values ratio, WT = wild-type, HET = heterozygous.

**Supplementary Fig. 3. Individual parametric [^11^C]SCH23390 *BP*_ND_ maps based on 90 min acquisition in three representative WT mice to depict failed voxels using MRTM.** Maps are generated using SRTM, MRTM, and Logan Ref, and they are overlaid onto the WT MRI template for anatomical localization. White arrowheads indicate failed voxels in the parametric maps obtained using MRTM. *BP*_ND_ = non-displaceable binding potential, WT = wild-type.

**Supplementary Table**

**Supplementary Table 1. Methodological comparison during the test-retest study of [^11^C]SCH23390 in WT mice and HET Q175DN littermates.**

|  | **Test** |  | **Retest** |  | ***p* value** |
| --- | --- | --- | --- | --- | --- |
|  | Mean (sem) |  | Mean (sem) |  |  |
| **Injected dose (MBq)** | | |  |  |  |
| WT | 4.6 (0.5) |  | 4.7 (0.5) |  | 0.67 |
| HET | 5.5 (0.5) |  | 5.4 (0.6) |  | 0.58 |
| **Molar activity (GBq/µmol)** |  |  |  |  |  |
| WT | 72.9 (2.7) |  | 70.0 (2.2) |  | 0.22 |
| HET | 74.3 (1.5) |  | 71.9 (1.5) |  | 0.06 |
| **body weight (g)** |  |  |  |  |  |
| WT | 30.9 (1.0) |  | 30.9 (1.3) |  | > 0.99 |
| HET | 26.9 (0.4) |  | 26.8 (0.4) |  | 0.36 |
| **injected mass (µg/kg)** |  |  |  |  |  |
| WT | 1.11 (0.16) |  | 1.25 (0.12) |  | 0.09 |
| HET | 1.39 (0.12) |  | 1.50 (0.16) |  | 0.18 |

sem = standard error of the mean. WT = wild-type; HET = heterozygous. WT, *n* = 4; HET, *n* = 7.
